# Three Dimensional Checkerboard Synergy Analysis of Colistin, Meropenem, Tigecycline against Multidrug-Resistant Clinical *Klebsiella pneumonia* Isolates

**DOI:** 10.1371/journal.pone.0126479

**Published:** 2015-06-11

**Authors:** Claudia Stein, Oliwia Makarewicz, Jürgen A. Bohnert, Yvonne Pfeifer, Miriam Kesselmeier, Stefan Hagel, Mathias W. Pletz

**Affiliations:** 1 Center for Infectious Diseases and Infection Control, Jena University Hospital, Erlanger Allee 101, D-07747, Jena, Germany; 2 Institute of Medical Microbiology, Jena University Hospital, Erlanger Allee 101, D-07747, Jena, Germany; 3 Nosocomial Pathogens and Antibiotic Resistance, Robert Koch Institute, Burgstrasse 37, D-8855, Wernigerode, Germany; 4 Clinical Epidemiology, Integrated Research and Treatment Center, Center for Sepsis Control and Care (CSCC), Jena University Hospital, Erlanger Allee 101, D-07747, Jena, Germany; Cornell University, UNITED STATES

## Abstract

The spread of carbapenem-non-susceptible *Klebsiella pneumoniae* strains bearing different resistance determinants is a rising problem worldwide. Especially infections with KPC (*Klebsiella pneumoniae* carbapenemase) - producers are associated with high mortality rates due to limited treatment options. Recent clinical studies of KPC-blood stream infections revealed that colistin-based combination therapy with a carbapenem and/or tigecycline was associated with significantly decreased mortality rates when compared to colistin monotherapy. However, it remains unclear if these observations can be transferred to *K*. *pneumoniae* harboring other mechanisms of carbapenem resistance. A three-dimensional synergy analysis was performed to evaluate the benefits of a triple combination with meropenem, tigecycline and colistin against 20 *K*. *pneumoniae* isolates harboring different β-lactamases. To examine the mechanism behind the clinically observed synergistic effect, efflux properties and outer membrane porin (Omp) genes (*ompK35* and *ompK36*) were also analyzed. Synergism was found for colistin-based double combinations for strains exhibiting high minimal inhibition concentrations against all of the three antibiotics. Adding a third antibiotic did not result in further increased synergistic effect in these strains. Antagonism did not occur. These results support the idea that colistin-based double combinations might be sufficient and the most effective combination partner for colistin should be chosen according to its MIC.

## Introduction

The global spread of extended-spectrum β-lactamase (ESBL) producing enterobacteria has resulted in a worldwide increase in carbapenem consumption. This selective pressure has fostered the emergence and spread of carbapenem hydrolyzing β-lactamase variants, like KPC, OXA-48, VIM or NDM in Gram-negative bacteria [1]. Of these, the class KPC-carbapenemase is most frequently associated with *K*. *pneumoniae* but also metallo-β-lactamases (MBLs) and the carbapenemase OXA-48 are regionally widespread in this species. The first *K*. *pneumoniae* infection associated with KPC was reported in 2001 in the USA [2]. Presently, KPC-producing *K*. *pneumoniae* strains have spread across the north-eastern USA and southern European countries. In northern European countries KPC have been endemically reported [3]. In contrast, the proportion of KPC in *K*. *pneumoniae* blood stream infections in southern European countries was recently reported with up to 49.8% (Greece) [3].

KPC infections are related with high mortality rates of > 50% [4, 5] and treatment options are often limited by further resistances to other classes of antibiotics, e.g. fluoroquinolones [6]. Besides KPC, other β-lactamases are frequently co-produced, e.g. TEM, SHV, CTX-M and OXA variants, resulting in a high resistance to all β-lactams. Moreover, usage of carbapenems triggers the occurrence of *K*. *pneumoniae* mutants that lose the major outer membrane porins OmpK35 and/or OmpK36. Loss of these porins is known to contribute to carbapenem resistance e.g. by increasing the minimal inhibitory concentration (MIC) of carbapenems in ESBL producers [7]. First outbreaks caused by carbapenem-non-susceptible *K*. *pneumoniae* with ESBLs (CTX-M-15 or SHV-12) and defective OmpK-porins have been recently reported from Italy [8], Portugal [9], and Greece [10].

In retrospective studies, reduced mortality rates were found among patients with various infections caused by KPC-producing *K*. *pneumoniae* under combination antimicrobial therapy when compared with patients receiving a monotherapy [11, 12]. In this context, the most frequent combinations were colistin or tigecycline plus a carbapenem. In one of these studies, the triple-drug combination, including tigecycline, colistin and meropenem, was associated with the highest survival rate [12]. The synergism of the colistin / tigecycline combination against various resistant *Enterobacteriacae* was demonstrated *in vitro* by checkerboard and time-killing studies and *in vivo* in a simple animal model (*Galleria mellonella*) [13]. However, the mechanism of this synergistic effect is still elusive.

Based on these previous studies, we performed *in vitro* experiments using a 3-dimensional checkerboard assay of colistin, tigecycline and meropenem primarily against clinical multidrug-resistant *K*. *pneumoniae* isolates harboring different β-lactamase types. Furthermore, we analyzed the efflux and the porin genes of these isolates to obtain an insight into the mechanisms of synergistic effects of this antimicrobial combination.

## Material and Methods

### Microorganisms and media

All clinical *K*. *pneumoniae* isolates (n = 20) used in this study were provided by the Robert Koch Institute (Wernigerode, Germany), the National Reference Laboratory for Multidrug-resistant Gram-negative Bacteria (Bochum, Germany) and the Institute of Medical Microbiology (Jena, Germany). All strains exhibited different antimicrobial susceptibility patterns and contained different β-lactamases and therefore are referred as non-clonal (Table 1). In order to establish the *K*. *pneumoniae* efflux phenotype, we also included 20 carbapenem-susceptible *K*. *pneumoniae* with an ESBL-phenotype that were retrieved from a current screening program in our institution (S1 Table). The MIC of each antibiotic was determined by broth microdilution technique in accordance to European Antimicrobial Susceptibility Testing Committee (EUCAST) standards [ISO 20776–1:2006]. Colistin (Sigma Aldrich, Germany), tigecycline (Sigma Aldrich, Germany) and meropenem (Hexal, Germany) were prepared as stock solutions of 100 mg/mL and stored in aliquots at—20°C. Test solutions were prepared immediately before usage. Cation-adjusted Mueller-Hinton (MH) broth (Carl Roth GmbH, Germany) or MH agar were prepared according to the manufacturer’s instructions.

**Table 1:** Results of susceptibility testing (MICs), the molecular analyses of β-lactamases and porins, the efflux properties (EHT), and the synergism testing (FICIs).

Strain*	MIC_MEM_ mg/L	MIC_TGC_ mg/L	MIC_CST_ mg/L	β-lactamase variants	Lost porin	EHT s	FICI_MEM/TGC_	FICI_MEM/CST_	FICI_TGC/CST_	FICI_MEM/TGC/CST_
RKI 536/13	48	4	16	KPC-2, SHV-11, TEM-1, OXA-9**, VEB-1, OXA-10	OmpK35 (stop), OmpK36 (frame shift)	70.4±4.0	**0.74±0.25 (0.4)**	**0.56±0.19 (0.4)**	**0.56±0.28 (0.4)**	**0.4±0.12 (0.2)**
NRZ 00246	64	8	16	OXA-48, CTX-M-15, OXA-1, TEM-1, SHV-1	OmpK36 (stop)	49.5±2.0	**0.75±0.18** (0.6)	**0.59±0.33 (0.2)**	**0.63±0.35 (0.3)**	**0.4±0.15 (0.4)**
RKI 178/11	8	4	16	CTX-M-15, TEM-1, SHV-11	OmpK36 (stop)	86.0±1.1	1.03±0.13 (0.8)	**0.64±0.25 (0.4)**	**0.55±0.28 (0.3)**	**0.61±0.2 (0.3)**
NRZ 04322	64	4	64	KPC-3, OXA-9**, TEM-1, SHV-11	OmpK35 (stop), OmpK36 (2 aa ins)	59.0±7.9	1.02±0.29 **(0.5)**	0.79±0.25 **(0.5)**	**0.56±0.35 (0.3)**	**0.69±0.33 (0.2)**
RKI 84/14	32	4	32	CTX-M-15, OXA-1, TEM-1, SHV-1	OmpK35 (stop), OmpK36::IS5-like	71.3±8.2	1.03±0.21 (0.6)	**0.66±0.43 (0.5)**	**0.53±0.29 (0.2)**	**0.66±0.37 (0.2)**
RKI 551/13	96	2	8	KPC-3,TEM-1, SHV-11, OXA-9**	OmpK35 (stop), OmpK36 (2 aa ins)	62.6±3.6	1.13±0.15 (0.9)	1.33±0.3 (1.0)	**0.53±0.23 (0.3)**	**0.81±0.36 (0.3)**
RKI 85/14	16	1	2	KPC-2, OXA-9**, TEM-1, SHV-12	OmpK35 (stop)	68.3±9.6	1.13±0.28 (1.0)	**0.65±0.3 (0.3)**	1.02±0.32 **(0.3)**	1.04±0.32 **(0.5)**
NRZ 08996	16	1	16	KPC-2, CTX-M-15, OXA-1, OXA-9**,TEM-1, SHV-1	OmpK36 (1 aa ins, 3 aa del, various aa sub)	73.3±16.3	1.06±0.5 (0.6)	1.13±0.72 (0.8)	1.0±0.23 (0.6)	**0.88±0.68** (0.6)
NRZ 01732a	256	1	16	SHV-11, VIM-1	OmpK35 (stop), OmpK36 (1 aa sub)	101.6±4.9	1.01±0.27 **(0.3)**	1.0±0.19 **(0.6)**	1.03±0.22 **(0.5)**	0.94±0.25 **(0.5)**
NRZ 01839	16	1	8	OXA-48, CTX-M-15, OXA-1, TEM-1, SHV-1	OmpK35 (stop), OmpK36 (3 aa ins, 3 aa del, various aa sub)	66.3±6.3	1.13±0.22 (1.0)	1.03±0.46 **(0.5)**	0.88±0.26 **(0.5)**	1.0±0.39 **(0.5)**
NRZ 02915	32	1	16	OXA-48, CTX-M-15, OXA-9, OXA-1, TEM-1, SHV-1	OmpK35 (stop), OmpK36 (3 aa ins, 3 aa del, various aa sub)	48.6±1.3	1.13±0.2 (1.0)	1.1±0.43 **(0.5)**	1.06±0.25 **(0.5)**	1.13±0.37 (0.6)
RKI 318/11	32	1	2	KPC-2, TEM-1, SHV-11, OXA-9**	OmpK35 (stop)	78.6±3.1	1.1±0.21 (1.0)	1.01±0.18 (0.6)	1.09±0.09 (1.0)	1.13±0.21 (0.6)
RKI 346/12	32	2	2	OXA-48, CTX-M-15, OXA-9, OXA-1, TEM-1, SHV-1	OmpK35 (stop), OmpK36 (3 aa ins, 3 aa del, various aa sub)	106.0±1.4	1.12±0.21 (1.0)	0.92±0.21 **(0.5)**	0.88±0.25 **(0.5)**	1.13±0.4 **(0.5)**
RKI 83/14	4	1	1	CTX-M-15, OXA-1, TEM-1, SHV-1	OmpK35 (stop), OmpK36::IS5-like	88.5±15.0	1.1±0.18 (1.0)	1.06±0.17 (0.8)	1.06±0.18 (0.6)	1.14±0.26 (0.6)
RKI 105/10	4	4	1	CTX-M-15, TEM-1, SHV-11, OXA-1	OmpK35 (stop), OmpK36 (stop)	71.0±1.3	1.04±0.1 (0.8)	1.04±0.15 (0.7)	1.13±0.15 (1.0)	1.14±0.19 (0.7)
NRZ 06142	64	1	16	KPC-2, VIM-1, TEM-1, SHV-11, OXA-9**	OmpK35 (stop), OmpK36 (frame shift)	57.6±10.1	1.04±0.15 (0.7)	1.0±0.31 **(0.5)**	1.09±0.27 (0.8)	1.16±0.31 (0.6)
NRZ 03656	32	0.5	16	OXA-48, CTX-M-15, OXA-9, OXA-1, SHV-1, TEM-1	OmpK35 (stop), OmpK36 (3 aa ins, del, various aa sub)	53.9±3.0	1.13±0.21 (1.0)	1.02±0.41 **(0.5)**	1.13±0.19 **(0.4)**	1.19±0.28 (0.7)
RKI 412/11	256	1	1	KPC-3, TEM-1, SHV-11, OXA-9**	OmpK35 (stop), OmpK36 (2 aa ins, 1 aa sub)	nm	1.11±0.2 (1.0)	1.02±0.26 (0.8)	1.13±0.31 (0.8)	1.2±0.29 (0.8)
RKI 60/11	4	1	0.5	CTX-M-15, TEM-1, SHV-11	OmpK35::IS3-IS911-like	72.9±12.3	1.12±0.16 (1.0)	1.03 ±0.15 (0.6)	1.25±0.28 (0.5)	1.29±0.17 (1.0)
NRZ 05989	48	0.5	1	KPC-2, SHV-1+12	OmpK35 (stop)	59.7±4.9	1.14±0.23 (1.0)	1.15±0.16 (1.0)	1.25±0.18 (1.1)	1.3±0.26 (0.8)

The EHT and the FICI values are given as median ± standard deviation. Synergistic FICI values are indicated in bold letters. The FICI values indicated in brackets represent the lowest values for the given combination.

*Strains were obtained from: NRZ = National Reference Laboratory for multidrug-resistant gram-negative bacteria, Ruhr-University Bochum, Germany, and RKI = Robert-Koch-Institut, Wernigerode, Germany; MEM = meropenem; CST = colistin; TCG = tigecycline; FICI = fractional inhibitory concentration index; EHT = efflux half-time; nm = not measureable; aa = amino acid;:: = gene dispuption by insertion sequence; del = deletion; ins insertion; sub = substitution;

**non-functional (STOP mutation in *bla*_OXA-9_ gene)

### Three dimensional checkerboard assays

**Fig 1:**
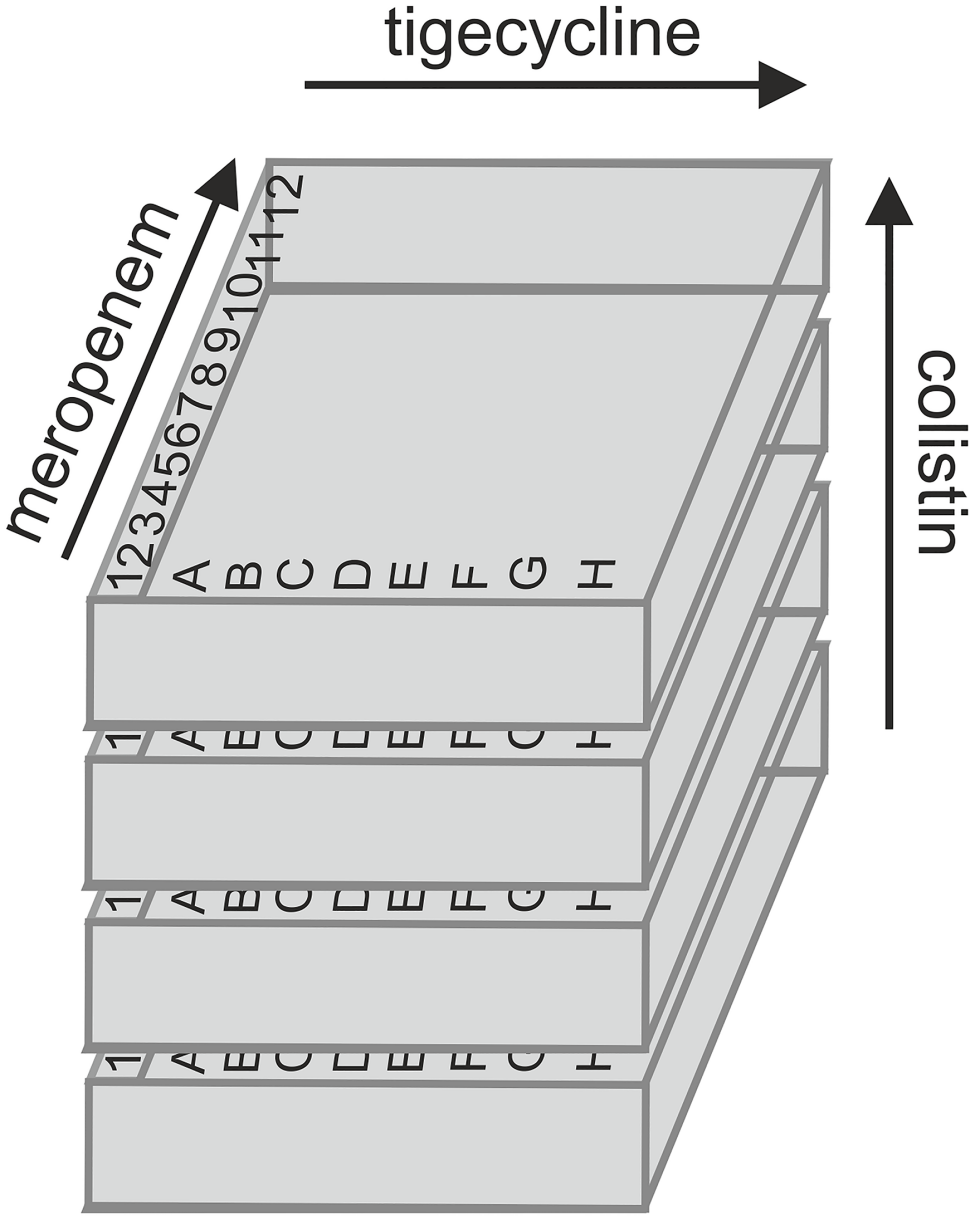
Schematic set-up of three-dimensional checkerboard technique. Concentrations of each drug increase towards the arrow. The FICI values were determined for each combination: meropenem / tigecycline, meropenem / colistin, tigecycline / colistin and meropenem / tigecycline / colistin.

Bacterial cells were cultivated overnight at 35°C at constant rotation (200 rpm) in MH broth. The overnight cultures were adjusted to 0.5 McFarland (equivalent to 10^8^ CFU/mL) and diluted 1:100 with broth to obtain a 10^6^ CFU/mL suspension. For the checkerboard assay, the broth microdilution method was modified by including some additional antibiotic concentrations. Into each well of a standard microwell plate 100 μL of the 10^6^ CFU/mL bacterial suspensions were transferred and mixed with an equal volume of antimicrobial solution. For each strain and antibiotic the selected concentration ranges depended on previously determined MICs. In total, 11 dilution steps of meropenem, 7 dilution steps of tigecycline and 6 dilution steps of colistin were analyzed (Fig 1). The microwell plates were incubated at 35°C for 16–20 h and interpreted according to EUCAST breakpoints for *Enterobacteriaceae*. Each test was performed at least in duplicate and included a growth control without addition of any antibiotic.

The fractional inhibitory concentration index (FICI) for each double (Eq 1) or triple (Eq 2) antibiotic combination was calculated as follows:

$$FICI_{A/B}=\frac{MIC_{A(combination)}}{MIC_{A(alone)}}+\frac{MIC_{B(combination)}}{MIC_{B(alone)}}$$ (1)

$$FICI_{A/B/C}=\frac{MIC_{A(combination)}}{MIC_{A(alone)}}+\frac{MIC_{B(combination)}}{MIC_{B(alone)}}+\frac{MIC_{C(combination)}}{MIC_{C(alone)}}$$ (2)

The FICIs were calculated using the concentrations in the first non-turbid well found in each row and column along the turbidity / non-turbidity interface and expressed as the median value. We used the median averaged FICI values instead of the lowest value to avoid over-interpretation of the synergism due to methodological (one-well) errors of the double dilution method. In this regard, we refer to the FICI interpretation proposed by [14]: FICI < 0.8 synergy, 0.8 < FICI < 4 additive effects or indifference, and FICI ≥ 4 antagonism. We recorded the lowest FICI values of the combinations (Table 1, indicated in brackets) as some authors prefer those, but we did not discuss these vales.

### Combined disk diffusion test

The disk diffusion test was preformed according to EUCAST rules for antimicrobial susceptibility testing. Bacterial suspensions of 0.5 McFarland were spread over the surface of the MH agar plates (diameter 90 mm) using a sterile swab. On sterile cellulose discs (diameter 6 mm) 10 μL of each antibiotic solution were applied to obtain 40 μg meropenem, 20 μg colistin and 15 μg tigecycline per disc. The three disks where placed at different distances to examine single and combination effects of the antibiotics on bacterial growth. Synergism was defined as extended edge of the inhibition zone of one antibiotic towards the disc of another antibiotic.

### Analysis of porin genes

The porin genes *ompK35* and *ompK36* were amplified and sequenced by using primers described previously [15]. The obtained nucleotide sequences were compared with wild-type *ompK* sequences of *K*. *pneumoniae* strain JM45 (accession number CP006656) available at the NCBI database.

### Efflux assay

A novel fluorescent arylidenehydantoin piperazine dye BM-27 was used for the assay (Bohnert JA and Handzlik, J, manuscript in preparation), which is virtually non-fluorescent in aqueous solution but fluoresces strongly upon binding to periplasmic phospholipids. BM-27 was developed in a set of N3- aminealkyl arylidenehydantoin derivatives [16] that are substrates and inhibitors of the MDR efflux pump AcrAB-TolC [17].

20 mL of the overnight cultures were centrifuged and washed twice with potassium phosphate buffer (PPB) containing 12.5 mM K_2_HPO_4_, 7.8 mM KH_2_PO_4_ and 1 mM MgCl_2_ (pH 7). The bacterial pellets were resuspended in PPB and adjusted to an optical density of 0.5 at 600 nm. The efflux assay was performed in flat, transparent 96-well plates (Greiner, Germany) using Infinite M200Pro spectrometer (Tecan, Switzerland). In each well 200 μL bacterial suspensions and 15 μM carbonyl cyanide 3-chlorophenylhydrazone (CCCP) (Sigma Aldrich) were mixed and incubated for 8 min at room temperature to disrupt the residual proton gradient of the cells. Bacterial cells were labeled with 50 μM of BM-27. Fluorescence intensity was determined at 400 nm excitation and 457 nm emission. The treatment time varied depending on the labeling of the respective strains and was completed by reaching a constant fluorescence signal (steady-state). By adding glucose to a final concentration of 50 mM the efflux of the BM-27 dye was initiated and the time-resolved decrease of the fluorescence signal was measured. The obtained curves could be best fitted by applying the general fit for one-phase exponential decay (Eq 3) using GraphPad Prism 6.00 (GraphPad Software, La Jolla California USA):

$$y=(F_{max}-F_{min})\cdot e^{-KX}+F_{min}$$ (3)

Where y is the measured fluorescence signal, x represents the time ordinate, F_max_ represents the fluorescence signal at x = 0; F_min_ represents the fluorescence signal at infinite time. The efflux half-time (EHT) corresponds to the term 0.6932/K and was used as ratio to compare the efflux properties. The labeling efficiency ΔF was given by fluorescence difference of F_max_ to F_min_.

### Statistical analyses

Differences between two groups (e.g. *ompK* mutation absent or present) for several semi-quantitative FICIs (FICI_MER/TGC_, FICI_MER/CST_, FICI_TGC/CST_ and FICI_MER/TGC/CST_) were investigated by exact non-parametric Wilcoxon-Mann-Whitney tests and quantified by median differences and 95% confidence intervals (CIs). Similarly we determined non-parametric Spearman’s rank correlation coefficients ρ (and 95% CIs) for semi-quantitative variables MICs and efflux with FICIs. We applied a two-sided significance level of 5% and report nominal two-sided p-values. Wilcoxon-Mann-Whitney tests were conducted using the statistical language R version 3.0.3 and the Spearman correlations were calculated using SAS version 9.3.

## Results

### Phenotypic and genotypic characterization of the resistances

All 20 analyzed clinical *K*. *pneumoniae* isolates harbored at least two various β-lactamases, including narrow-spectrum-β-lactamases (TEM-1, SHV-1, SHV-11 and/or OXA-11), ESBLs (CTX-M-15, OXA-1 and/or VEB-1) and carbapenemases (OXA-48, VIM-1 and/or KPC), resulting in different resistance profiles (according to EUCAST breakpoints) (Table 1). In three isolates additional non-functional OXA-9 genes were determined due to a nonsense mutation.

In total, 16 strains (80%) were meropenem-resistant (MIC_MEM_ > 8 mg/L). Within these, 5 showed susceptible or intermediate MICs for tigecycline (MIC_TGC_ ≤ 1 mg/L) and colistin (MIC_CST_ ≤ 2 mg/L), 7 were resistant to colistin (MIC_CST_ > 2 mg/L) and 4 were resistant to all three drugs. Four strains showed intermediate MICs for meropenem (MIC_MEM_ > 2–8 mg/L). Within these, one isolate was resistant and three were intermediate to tigecycline and colistin.

### Analysis of porin genes ompk35 and ompk36

**Fig 2:**
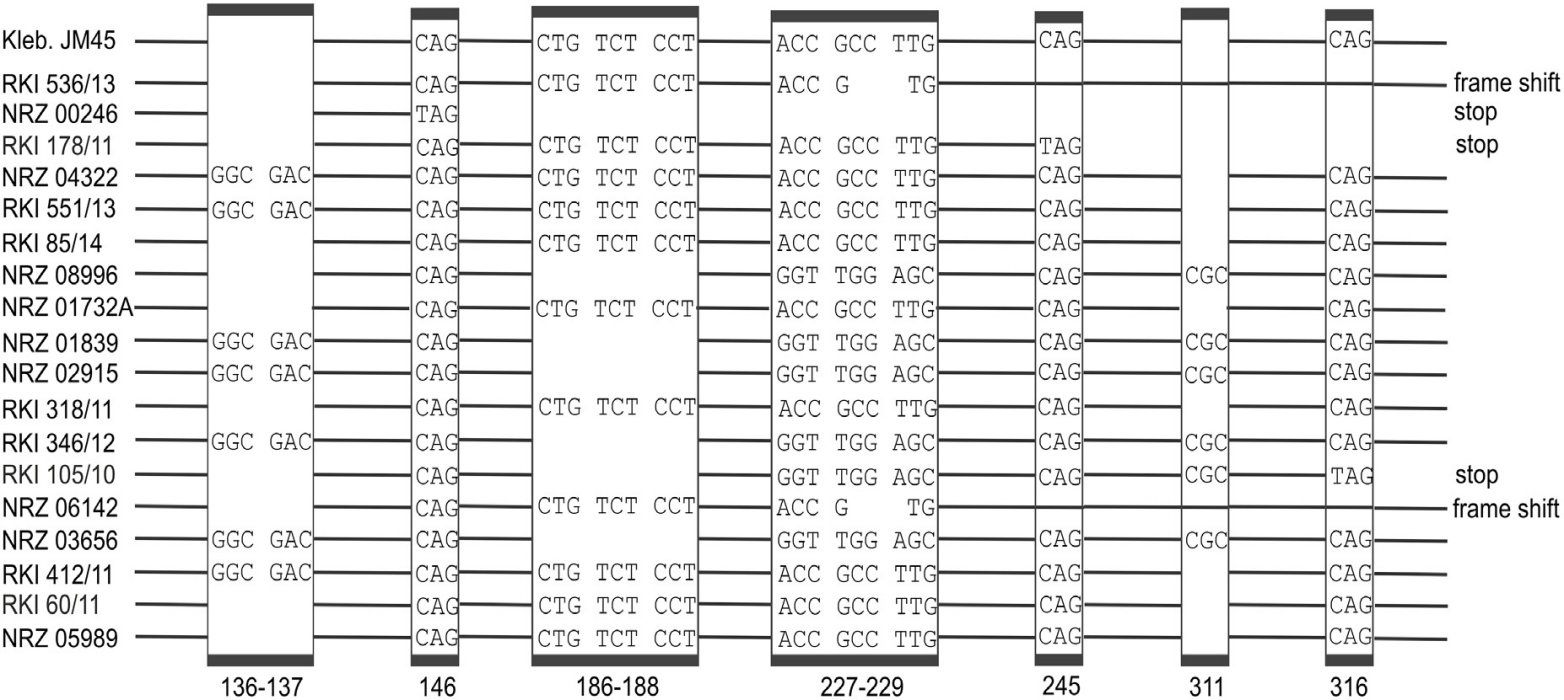
Sequence alignment of *ompK36* genes. The sequence of *ompK36* of *K*. *pneumoniae* strain JM45 (accession number CP006656) was used as reference. The boxes marked the most relevant changes within the *ompk36* sequences. Alterations are described above the boxes and the positions of the corresponding amino acid residues are indicated below the boxes.

Sequence analyses revealed that all strains exhibited mutations in the *ompK* genes. Three strains lost OmpK35 and three other strains lost OmpK36 due to nonsense mutations in the respective genes. One strain exhibited only a modified OmpK36. One strain lost both porins whereas 10 strains lost the OmpK35 but harbored modified OmpK36 (Fig 2).

The altered *ompK35* genes contained frame shift mutation due to one bp insertions (NRZ 04322, RKI 551/13, RKI 85/14, RKI 318/11, RKI 105/10, RKI 412/11, NRZ 05989) or one bp deletions (RKI 84/14, NRZ 01839, NRZ 02915, RKI 346/12, RKI 83/14, NRZ 03656), or one amino acid substitutions (RKI 536/13, NRZ 01732a, NRZ 06142) resulting in intergenic stop codons (data not shown) and consequently in early termination of the peptides. Alterations of gene *ompK36* were more variable. Two strains (RKI 84/14 and RKI 83/14) contained insertion sequences (*IS*5) within *ompK36* disrupting the open reading frame. Substitutions leading to stop codon TAG and early termination of OmpK36 were found at amino acid position 146 in NRZ 00246, at position 245 in RKI 178/11, and at position 316 in RKI 105/10. Two strains (RKI 536/13 and NRZ 06142) revealed deletions at amino acid position 228–229 leading to a frame shift with the consequence of prolonged peptide of unknown effect. Seven strains showed an insertion of 2 amino acids (glycine and aspartic acid) at positions 136 and 137 that are located within the highly conserved domain of loop structure L3 [18]. This mutation has been described to be associated with increased carbapenem MICs [19].

Further sequence modifications within the *ompK36* gene were found in some isolates (S2 Table) but the consequences for the function of the proteins were not studied in this work. Amino acid substitutions or in-frame insertions or deletions may lead to altered functions but are unlikely to result in total loss of the porin.

### Characterization of the efflux properties

The novel fluorescent dye BM-27 was used to measure the efflux properties of the strains. Contrary to other known membrane probes like Nile red [20], BM-27 is readily water soluble so no organic solvents with potentially antibacterial effects are needed.

Since no comparable data exist for *K*. *pneumoniae*, we first analyzed and interpreted the distribution of the maximal labeling efficiency by the fluorescent dye as well as the distribution of the efflux half-time (EHT) including ESBL *K*. *pneumoniae* isolates with meropenem susceptible phenotype. In general, it is more difficult to label *K*. *pneumoniae* strains with any dye compared to e.g. *Escherichia coli* due to the stronger outer membrane charge [21]. In the present study only one meropenem resistant strain (RKI 412/11) could not be suitably labeled for the efflux assay and therefore we cannot make any conclusions about its efflux properties. For the other strains labeling of > 900 fluorescence units were obtained and efflux curves were evaluable (S1 Fig). The influx and efflux distributions did not differed between meropenem resistant and susceptible isolates (p values 0.247 and 0.879, respectively). The influx properties seemed to be independent of the OmpK-porins since no correlation could be observed between ΔF and the respective porin mutation indicating that BM-27 might use other channels or mechanism to penetrate the cell wall.

EHT values were considered to interpret the efflux data. Most of the strains exhibited EHT values of 40–50 seconds (30%) or 50–60 seconds (35%), 15% showed EHT values of 60–70 seconds, whereas 5% showed EHT values of 70–80 seconds and 15% even higher values of 80–90 seconds (S1 Fig). Compared to other studies on *E*. *coli* efflux, 50–70 seconds correspond to moderate efflux whereas lower values indicate an enhanced efflux and higher values impaired efflux [20].

### Synergism testing

Except two isolates (RKI 536/13 harboring KPC-2 and NRZ 00246 harbouring OXA-48) that exhibit FICI_MEM/TGC_ of 0.74 and 0.75, no synergism was found for meropenem-tigecycline combination in all other strains (Table 1).

**Fig 3:**
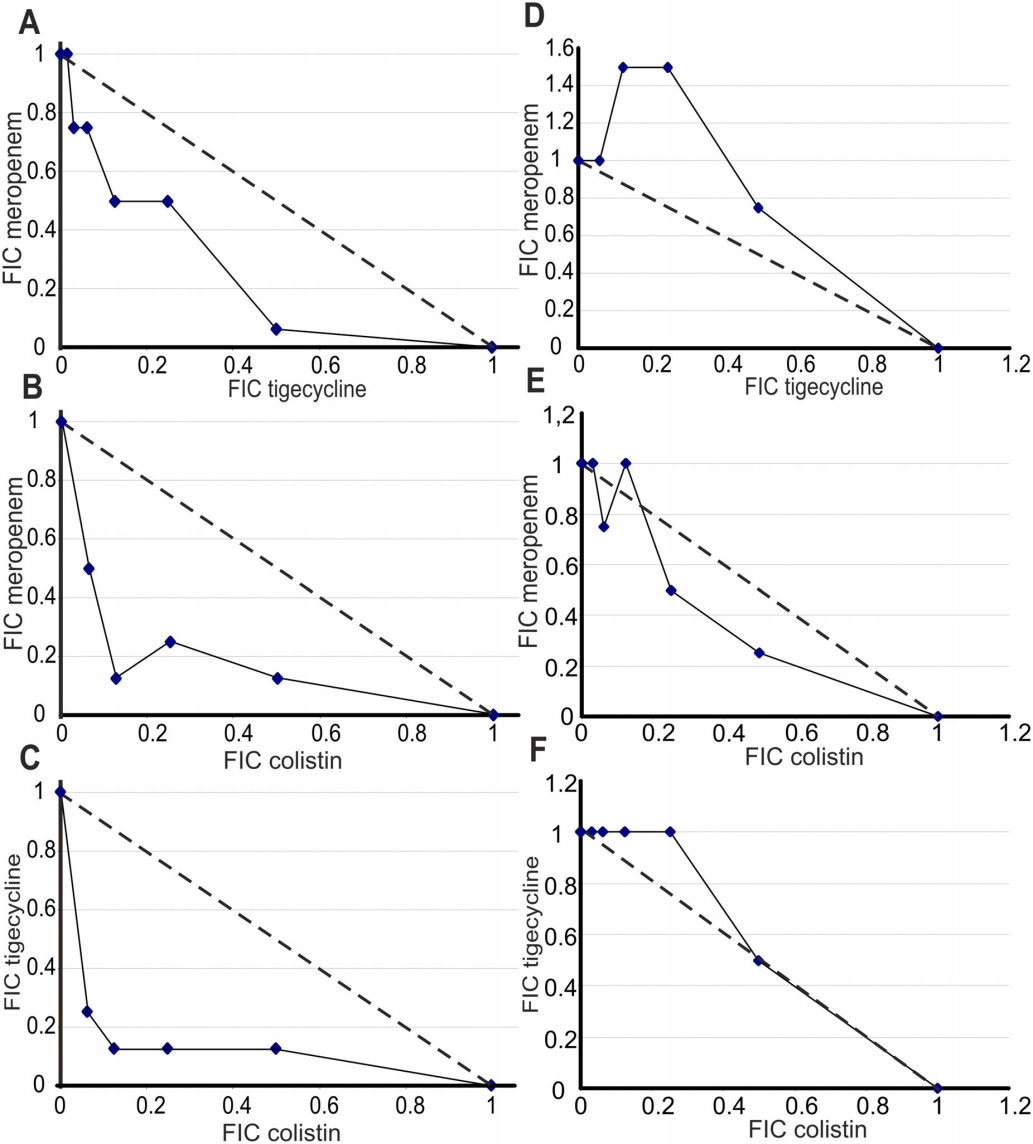
Exemplary isoboles of the double combinations of the antibiotics. A-C: Isoboles of strain NRZ 00246 (OXA-48 producer) exhibiting synergistic effects. D-F: Isoboles of strain RKI 318/11 (KPC producer) yielding no synergism. FIC = fractional inhibitory concentration.

Synergism of all three colistin-based combinations (MEM / CST, TGC / CST and MEM / TGC / CST) was found in 5 isolates (Fig 3A–3C). For one additional isolate, only MEM / CST and for another one TGC / CST and MEM / TGC / CST synergies were found. A clearly visible reduction of the FICI_MEM/TGC/CST_ compared to FICI_TGC/CST_ could not be observed for the isolates with synergism (Table 1) indicating that the addition of meropenem to the tigecycline / colistin combination or tigecycline to the meropenem / colistin combination did not increase the antimicrobial efficacy. No significant correlation of the MIC_MEM_ to the FICI_MEM/CST_ or FICI_MEM/TGC/CST_ could be determined. However, all isolates with FICI_MEM/CST_ < 0.8 exhibited MIC_MEM_ of ≥ 8 mg/L and carried various β-lactamase variants (KPC, OXA-48 or CTX-M-15) indicating that there is also no causal relationship between the β-lactamase type and synergism with colistin. The MICs for colistin showed some weak correlation with FICI_TGC/CST_ (ρ = -0.52, P = 0.017) and FICI_MEM/TGC/CST_ (ρ = -0.61, P = 0.003). Isolates with synergism generally showed increased MIC_CST_ values (MIC_CST_ > 8 mg/L). Similar correlations were found between increased resistance to tigecycline (MIC_TGC_) and the FICI_MEM/TGC_ (ρ = -0.70, P <0.001), FICI_TGC/CST_ (ρ = -0.70, P < 0.001) and FICI_MEM/TGC/CST_ (ρ = -0.73, P < 0.001). All isolates with synergism exhibited MIC_TGC_ ≥ 2 mg/L. A correlation between the efflux properties and the FICI values could not be shown. For detailed results see S3 Table.

**Fig 4:**
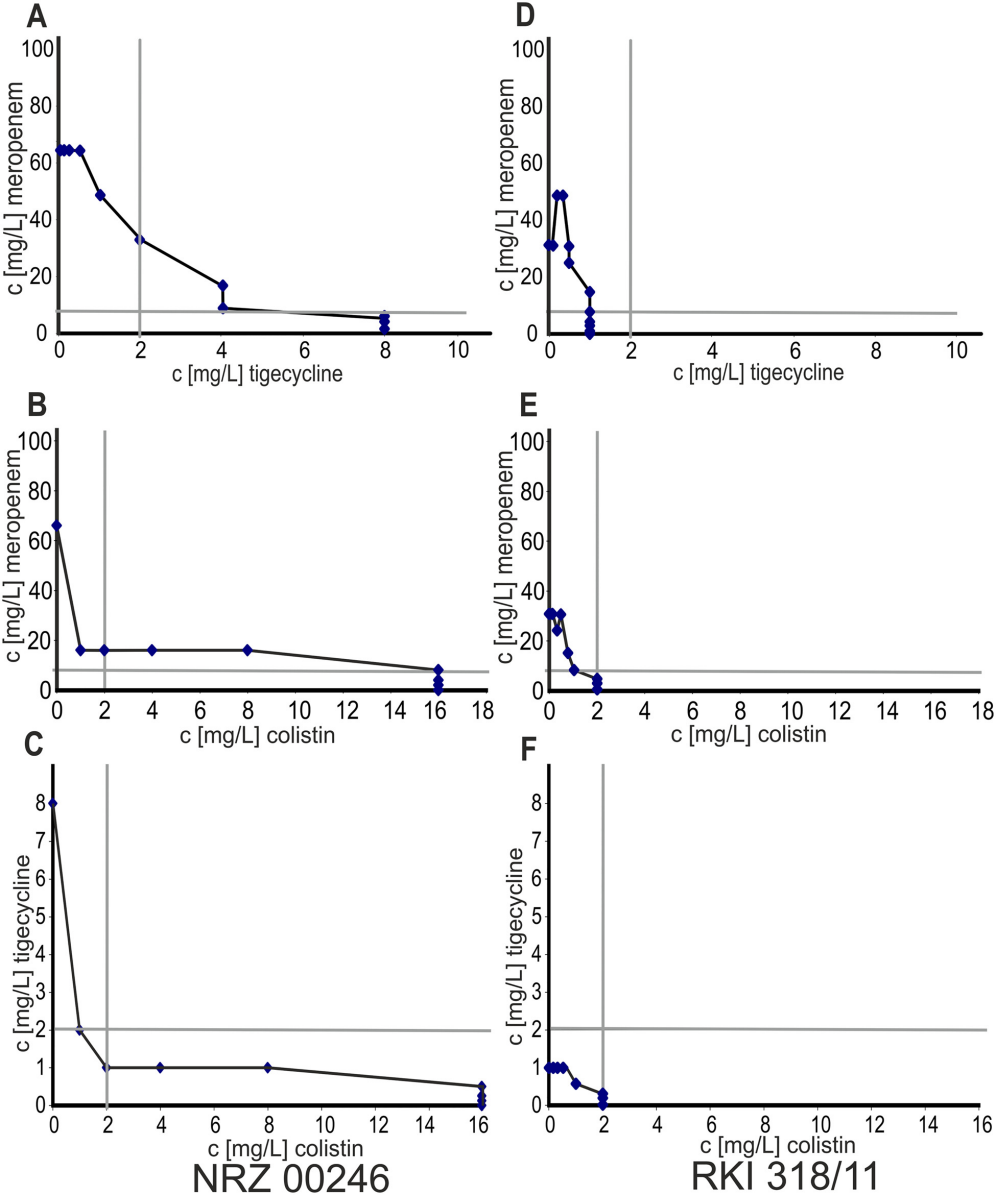
Exemplary plots of the checkerboard assays for the double combinations of the antibiotics. A-C: Strain NRZ 00246 (OXA-48 producer) exhibiting FICI <0.8. D-F: Strain RKI 318/11 (KPC producer) exhibiting FICI >0.8. Grey lines indicate the breakpoints of the respective antibiotic (according to EUCAST).

The MIC for at least one antibiotic could be strongly reduced in combination for highly resistant strains (Fig 4, S2 and S3 Figs): For the combination of colistin and tigecycline both MICs could be decreased below the EUCAST breakpoints.

Within the six isolates exhibiting synergistic effects (Table 1, FICIs in bold letters), four were found with loss of OmpK36 porin due to two non-senses mutations, one *IS*5-insertion and one frame shift mutation in the *ompK36* gene sequence, respectively. The two remaining isolates showed identical alterations of OmpK36 due to the insertion of glycine and aspartic acid in the L3 structure. Within these two isolates carried only narrow-spectrum and ESBL β-lactamases but non carbapenemase.

### Disk diffusion test

Disk diffusion tests were performed to supplement the results of the checkerboard assay. We selected 7 isolates exhibiting synergistic effects and 2 isolates that showed no synergism.

**Fig 5:**
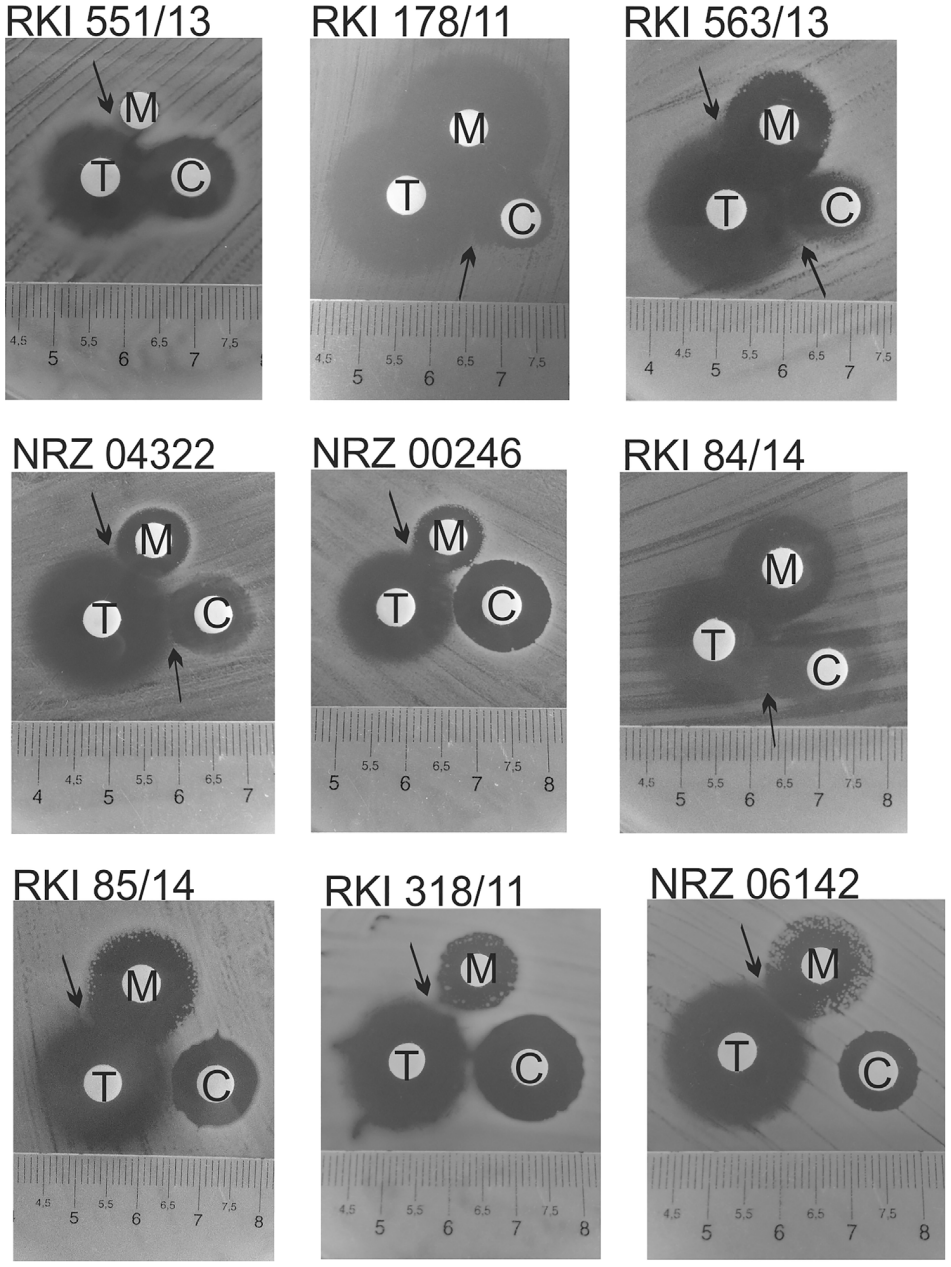
Inhibition zones of the antibiotics in disc diffusion test of selected strains.

Compared to the checkerboard test similar synergistic pattern were found for four strains (RKI 563/13, RKI 178/11, NRZ 04322 and RKI 551/13). For the strains NRZ 00246, RKI 84/14 and RKI 85/14 no clear synergism between colistin and the other antibiotics was visible, but synergistic effects of meropenem and tigecycline were found for these strains (Fig 5). Synergism of meropenem and tigecycline was also visible for two exemplary strains revealing no synergism in the checkerboard assay (NRZ 06142 and RKI 318/11). In this test, growth of mutants inside the inhibition zone of the respective antibiotic (in case of RKI 563/13: meropenem and colistin; RKI 85/14 and NRZ 06142: meropenem; and NRZ 04322 and RKI 84/14: colistin) was suppressed by the activity of the synergistic agonist tigecycline.

## Discussion

The number of *K*. *pneumoniae* isolates with reduced susceptibility to carbapenems increases annually and often this phenotype is carbapenemase independent. In the present study, to date rarely addressed porin defects were observed in all analyzed *K*. *pneumoniae* isolates (Table 1). Carbapenemase producers (KPC, OXA-48 or VIM-1) were consistently highly resistant to meropenem but also the MIC_MEM_ of five isolates harboring only ESBL variant CTX-M-15 or additionally the narrow-spectrum β-lactamase OXA-1 varied from 4 mg/L to 32 mg/L. The increased MICs for meropenem are most probably caused by the loss of the porins OmpK35 and/or OmpK36 as described previously [8, 22, 23]. Based on our results the proportional contribution of both OmpK-porins to the MIC_MEM_ remains unclear since no explicit pattern, due to the isolated loss of OmpK35 or OmpK36, could be evaluated. However, other studies demonstrated that both porins contribute to the carbapenem resistance in *K*. *pneumoniae* [24]. While loss of Omp35 alone was shown to have weak effects, OmpK36 is stronger associated with increased cephalosporine and carbapenem MICs of ESBL and AmpC producers and loss of both porins was shown to strongly elevate the resistance to most cephalosporines and carbapenems [22, 23]. Interestingly, isolate RKI 84/14 exhibit an exceptional high MIC_MEM_ of 32 mg/L. Since this isolate showed similar *omp*-mutation and similar β-lactamase variants compared to isolate RKI 83/14, the reduced meropenem susceptibility seems to be triggered by further unknown factors. In this context, loss of the porins LamB [25] or PhoE [26] that have been shown to contribute to carbapenem resistance or increased expression of the CTX-M gene might be involved but were not investigated in this work. The efflux properties of all study isolates could not been directly correlated to the MIC_MEM_ or MIC_CST_ or MIC_TGC_.

Using the checkerboard assay synergism of double and triple antibiotic combinations was identified in 30% of the *K*. *pneumoniae* isolates with phenotypic carbapenem resistance but was not significantly increased for the triple combination. Similar to a recently published work, no significant correlation between the synergistic effect of colistin / meropenem and loss or modifications of OmpK36 porin could be identified [27].

Retrospective clinical data indicated improved outcome for KPC *K*. *pneumoniae* infections for treatments including at least one drug with *in vitro* confirmed activity [28]. In the present study, synergistic effects were only observed for isolates with high MIC_MEM_ (> 8 mg/L) that simultaneously exhibited high MIC_CST_ (> 8 mg/L) and / or MIC_TGC_ (> 2 mg/L) and thus were classified as resistant to all three antibiotics. It has to be mentioned that the predictive value of *in vitro* antimicrobial susceptibility testing (AST) is often limited by various methodological and individual factors (focus of infection, co-morbidities or co-infections, antibiotic blood and tissue levels) [29]. Nevertheless, *in vitro* synergy testing might be helpful to elucidate the best-performing therapeutic partners. In this study the synergism was dominantly observed in colistin-based combinations, which may be explained as follows: The β-lactam antibiotics act within the periplasmatic space and primary pass though the outer membrane *via* the major porins OmpK35 and OmpK36 [30]. Tigecycline acts in the cytoplasm by inhibiting the 30S subunit of the ribosome [31] and has to cross both membranes. Colistin is a amphiphilic polymyxin and is known to interact with lipoid compounds and to induce instability and pore-formation in bacterial membranes [32], and thus it might promote the membrane translocation of meropenem and tigecycline. To overcome the increased resistance against periplasmatic or intracellular active antibiotics, higher concentrations of these antibiotics have to be achieved in the respective cell compartment. The colistin enhanced membrane translocation might be only realized in colistin resistant strains that can accumulate higher amounts of colistin without timely killing of the bacteria. This supports the idea that there is a relation between the permeability of the cell wall and membrane and the restored antimicrobial efficacy of meropenem and tigecycline even against resistant isolates by disregarding of the resistance mechanisms due to saturated antibiotic concentrations. A similar mechanism was proposed for the colistin-doripenem-ertapenem combination [27].

In the clinical context, synergism is only of value if the MICs of at least one combination partner are decreased below the respective breakpoint. Therefore we alternatively plotted MICs of the double antibiotic combinations (Fig 4) and compared the effective combined concentrations to EUCAST breakpoints. In our opinion these plots better illustrate the benefits of the respective combinations against individual strains. This allowed conclusions of the impact of each antibiotic to achieve an effective combination therapy. For example: Strain NRZ 00246 was meropenem, tigecycline and colistin resistant, and for all double combinations the FICIs indicated synergism. The simple plots clearly showed that meropenem combined with tigecycline (Fig 4A) or colistin (Fig 4B) reduced the required concentration of both antibiotics, but combined low concentrations of tigecycline and colistin were within the therapeutic window (and even within the breakpoints) of both antibiotics. Thus this combination seemed to be more effective (Fig 4C).

We could not determine synergistic effects in strains with a low MIC for one of the three tested antibiotics. It cannot be excluded that some synergism may become unnoticed due to the microdilution technique and the chosen concentration, but we favor the hypothesis that in susceptible strains one effective antibiotic is sufficient for growth inhibition *in vitro*. The plots in Fig 4D–4F demonstrate the distribution of the combined MICs in a susceptible strain, where no increase in FICI values was observed. Not surprisingly, all MICs are located below the breakpoints for the respective antibiotics.

Studies investigating colistin pharmacokinetic (PK) revealed that much higher dosages than the traditionally used 3 x 1 million units are required to achieve a serum concentration above 2 mg/L [33]. However, since colistin can cause serious side effects, increasing dosages is limited by toxicity [34]. In this study, we could show that even in colistin resistant strains a low colistin dosage (below 2 mg/L) is able to reduce the MIC or even restore the susceptibility against meropenem and tigecycline. The studied isolates were all defective in one or two major porins. These mutations are associated with increased carbapenem resistance in *K*. *pneumoniae* [7]. Thus most likely the synergistic mechanism is driven by colistin-induced facilitated translocation of meropenem and tigecycline though the outer and inner membranes. In line with our results, a recent retrospective analysis of carbapenemase-producing *K*. *pneumoniae* bloodstream infections did not find an advantage of the carbapenem-tigecycline combination but carbapenem-colistin and colistin-tigecycline combinations [35]. However, improved outcome of patients infected by *K*. *pneumoniae* under carbapenem-tigecycline combination therapy was observed in other retrospective studies [36, 37] suggesting that *in vitro* data might poorly reflect the *in vivo* efficiency. A combination therapy, even if not clearly synergistic in *in vitro* test, might be more effective than a monotherapy and prevent the emergence of resistant mutants as indicated by the disc diffusion assay in this study.

## Conclusion

Combination therapy offers a perspective to threat even highly resistant strains. Our *in vitro* results suggest that the best appropriate combination therapy to treat carbapenem resistant *K*. *pneumoniae* might be colistin / tigecycline. In highly resistant strains relevant synergism was detected resulting in a MIC that would have been clinically achievable by conventional dosing.

What do our observations mean for current clinical practice? Colistin seems to be the most effective part of these combinations. Antagonism even in triple combinations is unlikely. Even low colistin concentrations (< 2 mg/L) were able to restore meropenem and tigecycline susceptibility. Therefore, sufficient dosing of colistin may be more important than adding a third drug to a double combination. Adding a third antibiotic to a colistin based double combination might be only useful *in vivo* if the MIC of colistin is elevated but data confirming these hypothesis are elusive.

## Supporting Information

S1 Fig Results of the efflux assay. Distribution of the labeling efficiency (upper diagram) indicated as relative intracellular fluorescence uptake, and efflux properties (lower diagram) expressed as EHT values in seconds for *K*. *pneumoniae* isolates.
(TIF)

S2 Fig Plots of the combined MICs of the checkerboard assays for the antibiotic double combinations tested against isolates RKI 536/13, RKI 178/11, NRZ 04322, RKI 84/12, RKI 551/13, RKI 85/14. Isolates are indicated below the diagrams. Grey lines indicate the breakpoints of the respective antibiotic (according to EUCAST).
(TIF)

S3 Fig Plots of the combined MICs of the checkerboard assays for the antibiotic double combinations tested against isolates NRZ 08996, NRZ 01732a, NRZ01839, NRZ 02915, NRZ 06142, RKI 346/12, NRZ 03656. Isolates are indicated below the diagrams. Grey lines indicate the breakpoints of the respective antibiotic (according to EUCAST).
(TIF)

S1 Table Carbapenem-susceptible *K*. *pneumoniae* isolates with an ESBL-phenotype used as control group for the efflux assay. (DOCX)

S2 Table Further amino acid substitutions found in the OmpK36 variants. The translation of *ompK36* of *K*. *pneumoniae* strain JM45 (accession number CP006656) was used as reference sequence.
(DOCX)

S3 Table Statistical analysis of the correlation between various parameters. FICI values of double and triple combinations and MICs, ß-lactamases variants, OmpK mutations and influx/efflux properties. The correlations are calculated for the FICI medians and the lowest FICI values.
(DOCX)
