# Supplementary figures and images for: Three Dimensional Checkerboard Synergy Analysis of Colistin, Meropenem, Tigecycline against Multidrug-Resistant Clinical Klebsiella pneumonia Isolates
Source: PLoS One. 2015 Jun 11;10(6):e0126479. doi: 10.1371/journal.pone.0126479 (PMC4465894; doi:10.1371/journal.pone.0126479)

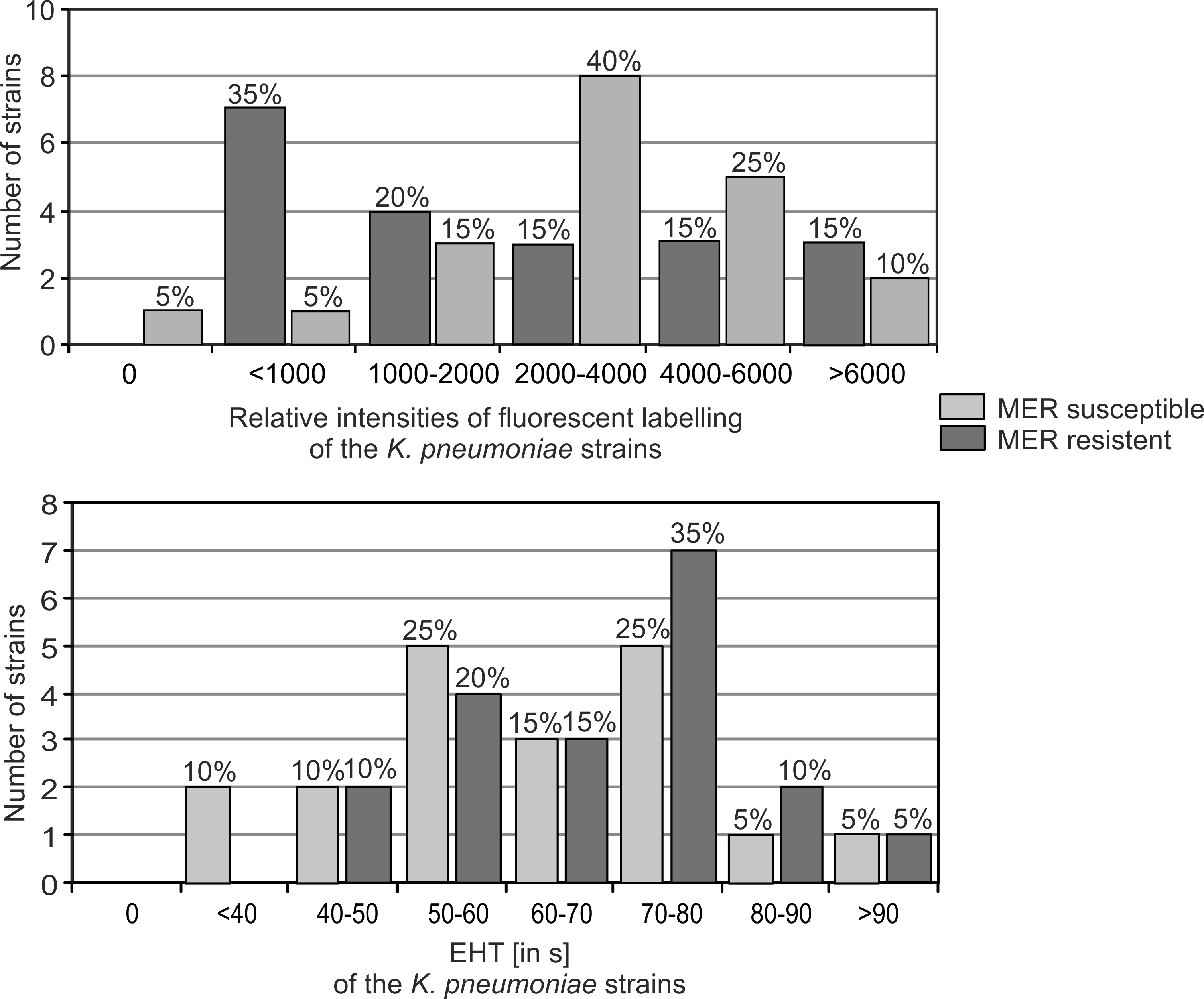

Supplement: S1 Fig — Distribution of the labeling efficiency (upper diagram) indicated as relative intracellular fluorescence uptake, and efflux properties (lower diagram) expressed as EHT values in seconds for K. pneumoniae isolates. (TIF) [file pone.0126479.s001.tif]

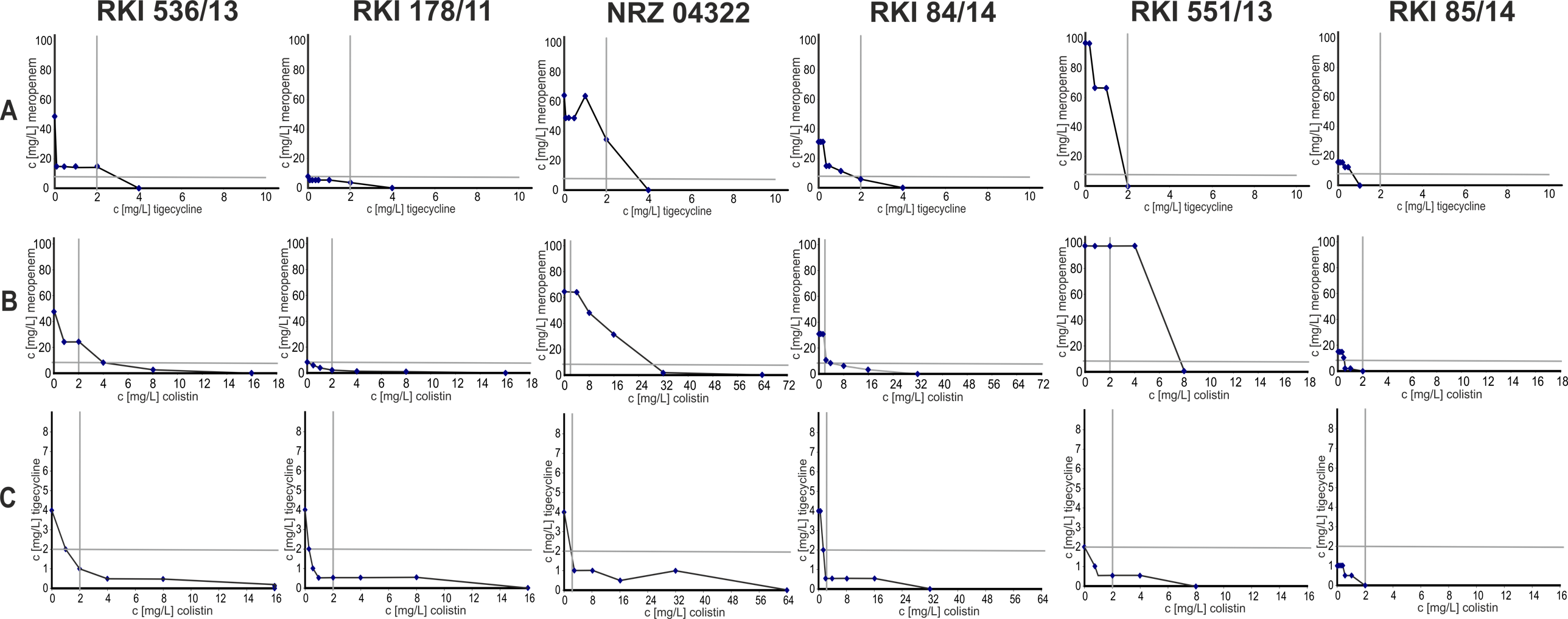

Supplement: S2 Fig — Isolates are indicated below the diagrams. Grey lines indicate the breakpoints of the respective antibiotic (according to EUCAST). (TIF) [file pone.0126479.s002.tif]

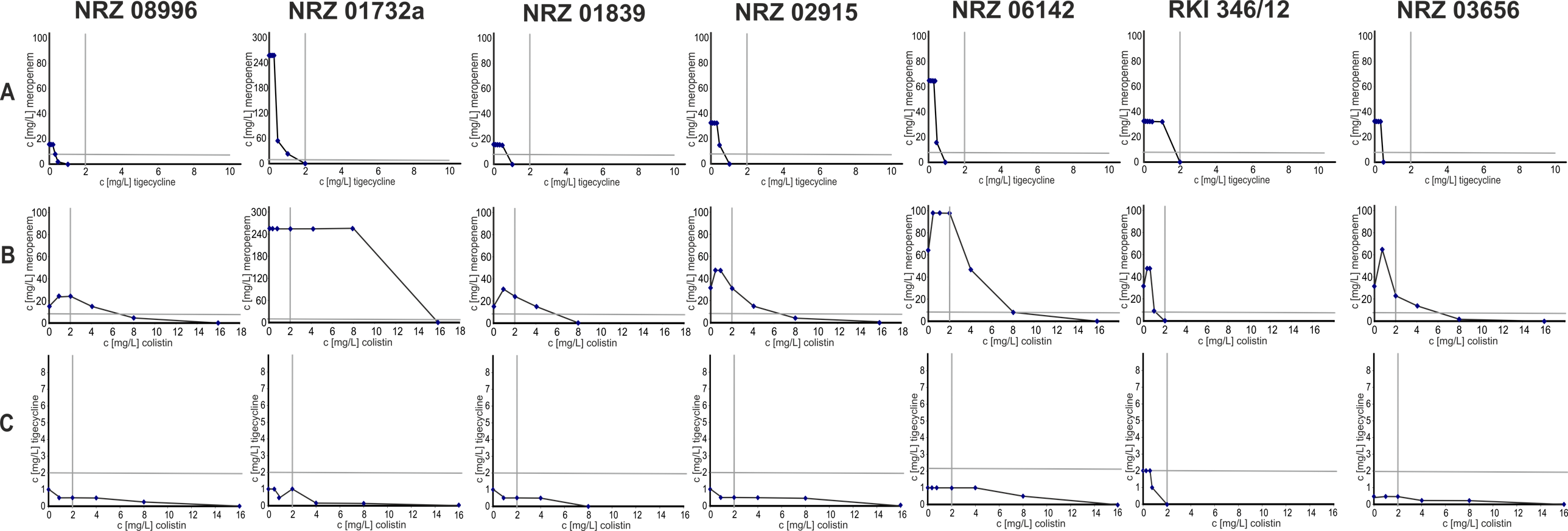

Supplement: S3 Fig — Isolates are indicated below the diagrams. Grey lines indicate the breakpoints of the respective antibiotic (according to EUCAST). (TIF) [file pone.0126479.s003.tif]
