# Supplementary material for: Three Dimensional Checkerboard Synergy Analysis of Colistin, Meropenem, Tigecycline against Multidrug-Resistant Clinical Klebsiella pneumonia Isolates
Source: PLoS One. 2015 Jun 11;10(6):e0126479. doi: 10.1371/journal.pone.0126479 (PMC4465894; doi:10.1371/journal.pone.0126479)
Supplement: S1 Table — (DOCX) [file pone.0126479.s004.docx]

**S1 Table. Carbapenem-susceptible *K. pneumoniae* isolates with an ESBL-phenotype used as control group for the efflux assay.**

| Strain | MIC_MEM_  mg/L | MIC_TGC_  mg/L | EHT  s |
| --- | --- | --- | --- |
| 2154 | <=0.25 | 1 | 70.77 |
| 2149 | <=0.25 | 2 | 70.36 |
| 1741/2 | <=0.25 | <=0.5 | 57.97 |
| 1974 | <=0.25 | 2 | 495.7 |
| 1453 | <=0.25 | 1 | 58.43 |
| 1265/2 | <=0.25 | <=0.5 | 61.64 |
| 217 | <=0.25 | 1 | 54.54 |
| 1956 | <=0.25 | <=0.5 | 40.21 |
| 1415 | <=0.25 | 1 | 31.91 |
| 1128/2 | <=0.25 | 2 | 39.74 |
| 942 | <=0.25 | 1 | 71.78 |
| 413/1 | <=0.25 | 2 | 51.48 |
| 833 | <=0.25 | 1 | 58.06 |
| 1030/2 | <=0.25 | >=8 | 90.34 |
| 1079 | <=0.25 | 2 | 60.65 |
| 2314 | <=0.25 | 2 | 72.75 |
| 1393 | <=0.25 | 1 | 65.07 |
| 1906 | <=0.25 | <=0.5 | 41.82 |
| 2308 | <=0.25 | 2 | 75.57 |
| 2092 | <=0.25 | 1 | 80.07 |

EHT = efflux half-time
