# Supplementary material for: Three Dimensional Checkerboard Synergy Analysis of Colistin, Meropenem, Tigecycline against Multidrug-Resistant Clinical Klebsiella pneumonia Isolates
Source: PLoS One. 2015 Jun 11;10(6):e0126479. doi: 10.1371/journal.pone.0126479 (PMC4465894; doi:10.1371/journal.pone.0126479)
Supplement: S2 Table — The translation of ompK36 of K. pneumoniae strain JM45 (accession number CP006656) was used as reference sequence. (DOCX) [file pone.0126479.s005.docx]

**S2 Table. Further amino acid substitutions found in the OmpK36 variants.** The translation of *ompK36* of *K. pneumoniae* strain JM45 (accession number CP006656) was used as reference sequence.

| Strain | Val180Pro | Asn191Arg | Tyr203Phe | Tyr212Trp | Asn223His | Leu227Thr | Gly228Asp | Asp229Glu | Asp230Glu | Lys233Val | Leu234Pro | Thr260Ser |
| --- | --- | --- | --- | --- | --- | --- | --- | --- | --- | --- | --- | --- |
| charge | nonpolar to nonpolar | polar to  basic | polar to nonpolar | polar to nonpolar | polar to basic | nonpolar to polar | polar to acidic | acidic to acidic | acidic to acidic | basic to nonpolar | nonpolar to nonpolar | polar to  polar |
| RKI 318/11 |  |  |  |  |  |  |  |  |  |  |  |  |
| RKI 412/11 |  | + |  |  |  |  |  |  |  |  |  |  |
| RKI 178/11 | + |  |  |  |  |  |  |  |  |  |  |  |
| RKI 60/11 |  |  |  |  |  |  |  |  |  |  |  |  |
| RKI 105/10 |  |  | + | + | + | + | + | + | + | + | + | + |
| RKI 83/14 |  |  |  |  |  |  |  |  |  |  |  |  |
| RKI 84/14 |  |  |  |  |  |  |  |  |  |  |  |  |
| RKI 85/14 |  |  |  |  |  |  |  |  |  |  |  |  |
| RKI 346/12 |  |  | + | + | + | + | + | + | + | + | + | + |
| RKI 536/13 | + |  |  |  |  |  |  |  |  |  |  |  |
| RKI 551/13 |  |  |  |  |  |  |  |  |  |  |  |  |
| NRZ 00246 |  |  | + | + | + | + | + | + | + | + | + | + |
| NRZ 01732a | + |  |  |  |  |  |  |  |  |  |  |  |
| NRZ 01839 |  |  | + | + | + | + | + | + | + | + | + | + |
| NRZ 03656 |  |  | + | + | + | + | + | + | + | + | + | + |
| NRZ 04322 |  |  |  |  |  |  |  |  |  |  |  |  |
| NRZ 06142 | + |  |  |  |  |  |  |  |  |  |  |  |
| NRZ 08996 |  |  | + | + | + | + | + | + | + | + | + | + |
| NRZ 02915 |  |  | + | + | + | + | + | + | + | + | + | + |
| NRZ 05989 |  |  |  |  |  |  |  |  |  |  |  |  |
