# Supplementary material for: Three Dimensional Checkerboard Synergy Analysis of Colistin, Meropenem, Tigecycline against Multidrug-Resistant Clinical Klebsiella pneumonia Isolates
Source: PLoS One. 2015 Jun 11;10(6):e0126479. doi: 10.1371/journal.pone.0126479 (PMC4465894; doi:10.1371/journal.pone.0126479)
Supplement: S3 Table — FICI values of double and triple combinations and MICs, ß-lactamases variants, OmpK mutations and influx/efflux properties. The correlations are calculated for the FICI medians and the lowest FICI values. (DOCX) [file pone.0126479.s006.docx]

**S3 Table. Statistical analysis of the correlation between various parameters.** FICI values of double and triple combinations and MICs, ß-lactamases variants, OmpK mutations and influx/efflux properties. The correlations are calculated for the FICI medians and the lowest FICI values.

|  | **FICI_MEM/TGC_** | | **FICI_MEM/CST_** | | **FICI_TGC/CST_** | | **FICI_MEM/TGC/CST_** | |
| --- | --- | --- | --- | --- | --- | --- | --- | --- |
|  | median | lowest | median | lowest | median | lowest | median | lowest |
| MIC_MEM_  [ρ (95% CI; p value)] | -0.17 (-0.57 to 0.30; 0.473) | -0.39 (-0.71 to 0.07; 0.086) | -0.13 (-0.54 to 0.33; 0.580) | 0.06 (-0.39 to 0.49; 0.804) | -0.20 (-0.59 to 0.27; 0.393) | -0.20 (-0.59 to 0.27; 0.395) | -0.22 (-0.60 to 0.25; 0.350) | -0.34 (-0.67 to 0.13; 0.149) |
| MIC_TGC_  [ρ (95% CI; p value)] | **-0.70 (-0.87 to -0.36; <0.001)** | **-0.56 (-0.80 to -0.15; 0.008)** | **-0.54 (-0.79 to -0.12; 0.012)** | -0.44 (-0.73 to 0.01; 0.051) | **-0.70 (-0.87 to -0.36; <0.001)** | **-0.63 (-0.83 to -0.25; 0.002)** | **-0.73 (-0.88 to -0.41; <0.001)** | **-0.69 (-0.86 to -0.34; 0.001)** |
| MIC_CST_  [ρ (95% CI; p value)] | **-0.45 (-0.74 to 0.00; 0.045)** | **-0.69 (-0.86 to -0.34; 0.001)** | -0.42 (-0.72 to 0.04; 0.067) | -0.40 (-0.71 to 0.06; 0.083) | **-0.52 (-0.78 to -0.09; 0.017)** | **-0.48 (-0.75 to -0.03; 0.032)** | **-0.61 (-0.82 to -0.22; 0.003)** | **-0.61 (-0.82 to -0.21; 0.004)** |
| influx  [ρ (95% CI; p value)] | -0.27 (-0.64 to 0.22; 0.267) | -0.37 (-0.70 to 0.12; 0.124) | -0.36 (-0.69 to 0.13; 0.137) | **-0.49 (-0.76 to -0.03; 0.034)** | -0.11 (-0.53 to 0.37; 0.661) | -0.19 (-0.59 to 0.29; 0.435) | -0.17 (-0.58 to 0.31; 0.494) | -0.15 (-0.56 to 0.33; 0.548) |
| efflux  [ρ (95% CI; p value)] | -0.19 (-0.59 to 0.29; 0.436) | 0.02 (-0.44 to 0.47; 0.952) | 0.08 (-0.39 to 0.51; 0.757) | 0.19 (-0.29 to 0.59; 0.442) | -0.08 (-0.51 to 0.39; 0.754) | 0.06 (-0.41 to 0.50; 0.808) | -0.01 (-0.46 to 0.45; 0.972) | 0.04 (-0.43 to 0.48; 0.886) |
| KPC  [median difference (95% CI; p value)] | 0.00 (-0.06 to 0.09; 0.512) | -0.01 (-0.25 to 0.22; 0.694) | 0.02 (-0.08 to 0.32; 0.724) | 0.04 (-0.12 to 0.30; 0.403) | 0.01 (-0.19 to 0.25; 0.866) | 0.04 (-0.25 to 0.31; 0.539) | 0.00 (-0.26 to 0.22; 1.00) | -0.03 (-0.27 to 0.22; 0.896) |
| OXA-48  [median difference (95% CI; p value)] | 0.02 (-0.02 to 0.09; 0.237) | 0.06 (-0.01 to 0.44; 0.156) | -0.03 (-0.23 to 0.27; 0.454) | -0.14 (-0.40 to 0.03; 0.123) | -0.03 (-0.38 to 0.33; 0.884) | -0.01 (-0.38 to 0.25; 0.884) | 0.00 (-0.22 to 0.39; 0.986) | -0.01 (-0.26 to 0.29; 0.946) |
| VIM-1  [median difference (95% CI; p value)] | -0.08 (-0.14 to 0.27; 0.300) | -0.34 (-0.70 to 0.14; 0.095) | -0.01 (-0.15 to 0.38; 0.763) | -0.02 (-0.49 to 0.28; 0.868) | 0.06 (-0.22 to 0.55; 0.605) | 0.16 (-0.50 to 0.50; 0.700) | 0.02 (-0.27 to 0.54; 0.747) | 0.00 (-0.29 to 0.34; 0.937) |
| CTX-M-15  [median difference (95% CI; p value)] | 0.01 (-0.07 to 0.12; 0.864) | 0.01 (-0.14 to 0.34; 0.315) | 0.00 (-0.29 to 0.17; 0.956) | -0.04 (-0.28 to 0.16; 0.502) | -0.02 (-0.25 to 0.31; 0.854) | -0.02 (-0.38 to 0.25; 0.852) | -0.01 (-0.26 to 0.25; 0.97) | 0.06 (-0.19 to 0.30; 0.539) |
| *ompK35* Stop  [median difference (95% CI; p value)] | 0.08 (-0.03 to 0.38; 0.118) | 0.23 (-0.22 to 0.44; 0.192) | 0.24 (-0.12 to 0.42; 0.406) | 0.16 (-0.22 to 0.44; 0.296) | 0.25 (-0.09 to 0.55; 0.167) | 0.19 (-0.12 to 0.69; 0.347) | **0.40 (0.01 to 0.73; 0.039)** | 0.19 (-0.10 to 0.46; 0.365) |
| *ompK36* Stop  [median difference (95% CI; p value)] | **-0.09 (-0.32 to -0.02; 0.004)** | **-0.22 (-0.44 to 0.00; 0.045)** | -0.27 (-0.41 to 0.03; 0.100) | -0.15 (-0.33 to 0.08; 0.101) | -0.19 (-0.50 to 0.06; 0.266) | -0.11 (-0.34 to 0.22; 0.547) | -0.24 (-0.60 to 0.02; 0.176) | -0.2 (-0.37 to 0.06; 0.341) |
